# 584 Major Outcomes After Chemical Injury from the American Burn Association Burn Research Dataset

**DOI:** 10.1093/jbcr/iraf019.213

**Published:** 2025-04-01

**Authors:** Jordan Garcia, Arman Fijany, Emily Swafford, Tyler Murphy, Punit Vyas, Robel Beyene, Stephen Gondek, Anne Wagner, Elizabeth Slater

**Affiliations:** Vanderbilt University Medical Center; Vanderbilt University Medical Center; Vanderbilt Burn Center; Vanderbilt Burn Center; Vanderbilt University Medical Center; Vanderbilt University Medical Center; Vanderbilt University Medical Center; Vanderbilt University Medical Center; Vanderbilt University Medical Center

## Abstract

**Introduction:**

Chemical burns often cause continuous tissue damage beyond their initial exposure, increasing the complexity of managing this type of burn injury. While the principles of management of chemical burns are similar to those for thermal burns, conventional management additionally involves prompt decontamination and extensive irrigation. This study investigates the outcomes for individuals with chemical burn injuries compared to those with non-chemical burn injuries.

**Methods:**

The American Burn Association (ABA) Burn Research Dataset was queried for admissions from 2012-2021 related to chemical or corrosion burns. The primary outcome assessed was mortality. Secondary outcomes were deep venous thromboembolism (DVT), hospital length of stay (LOS), ICU utilization, pneumonia, and respiratory failure. Minor complications included cellulitis, unplanned intubation, systemic sepsis, renal failure, cardiac arrest, and arrhythmia. A multivariate regression analysis was performed while controlling for the following covariates (age, sex, TBSA%, and inhalation injury) for these outcomes. Chi-square analysis was performed to analyze demographic and minor complication associations. Data analysis was done within the Python Language with PyCharm 3.1 software using the pandas and scipy.stats modules.

**Results:**

Of the 285,544 admissions within the dataset, 8,719 (3.1%) were related to chemical or corrosion burns. Admissions due to chemical burns were significantly more likely to be male (p < 0.05) and white (p < 0.05). Patients with chemical burns were significantly less likely to have acute respiratory distress syndrome (ARDS, p < 0.05). After adjusting for covariates, those with chemical burns had significantly lower mortality rates (p < 0.005) and ICU admission (p < 0.001). Admissions for chemical burns were also associated with a roughly 1.02-day shorter hospital LOS (p < 0.001). There were no significant differences for all other major and minor complications.

**Conclusions:**

Chemical burns represent a small percentage of burn injuries in the US. This study shows that chemical burns were associated with shorter hospital stays and decreased rates of ARDS, mortality, and ICU utilization compared to non-chemical burn injuries. Further research is required to understand long-term outcomes for individuals with chemical burn injuries as well as how initial management of these burns impacts clinical outcomes.

**Applicability of Research to Practice:**

Given that the mechanism and pathophysiology of chemical burns is distinct compared to non-chemical burn injuries, individuals with these injuries may require more specific assessment tools to better understand initial management and long-term sequelae of chemical burn injuries.

**Funding for the Study:**

N/A

